# Granulomatous Pancreatitis in a Patient with Acute Manifested Insulin-Dependent Diabetes Mellitus

**DOI:** 10.1155/2014/615426

**Published:** 2014-03-05

**Authors:** Václav Mandys, Michal Kheck, Michal Anděl

**Affiliations:** ^1^Department of Pathology, Third Faculty of Medicine, Charles University in Prague, 100 00 Prague 10, Czech Republic; ^2^Department of Pathology, Regional Hospital Jihlava, 586 33 Jihlava, Czech Republic; ^3^Second Department of Internal Medicine, Third Faculty of Medicine, Charles University in Prague, 100 00 Prague, Czech Republic

## Abstract

Isolated granulomatous noncaseating pancreatitis is a rare condition exceptionally described in human population. We demonstrate a case of the a 71-years-old female patient suffering from recent diabetes mellitus, generalized atherosclerosis and hypertension who died due to pulmonary embolism and terminal bronchopneumonia. Lipomatosis of pancreatic tissue was observed during the postmortem examination. Histological examination of pancreatic tissue discovered multiple small noncaseating epithelioid cell and giant cell granulomas, partly replacing the islets of Langerhans. To our knowledge, our case represents the first description of noninfectious granulomatous pancreatitis associated with acute manifested insulin-dependent diabetes mellitus.

## 1. Introduction

Noncaseating granulomatous inflammation confined to the pancreas has been only exceptionally described in human patients. Infections like tuberculosis and syphilis, exogenous noxes, autoimmunity, and systemic granulomatous diseases are the most frequent causes of granuloma formation within the pancreatic tissue [1]. Abdominal pain or epigastric discomfort, diarrhoea, weight loss, and obstructive jaundice are listed among the clinical symptoms of granulomatous pancreatitis [1–3]. We report a case of a patient who clinically presented with acute manifested diabetes mellitus associated with isolated granulomatous pancreatitis discovered in the postmortem examination and we present a review of the available literature.

## 2. Clinical History

A 71-year-old obese woman was admitted with the recent onset of diabetes mellitus manifested as hyperglycaemic ketoacidotic precoma. The past medical history was unremarkable. Recently, arterial hypertension was discovered. Her body weight was 110 kg, BMI 38. The plasma glucose level ranged from 3.1 to 15.1 mmol/L. The patient was treated with intensified insulin regime. The status of the patient was complicated by intermittent fever and several antibiotics were repeatedly administered. Terminally, clinical signs of septic shock and multiorgan failure appeared and the patient died. Postmortem examination performed 11 hours after death discovered signs of septic shock with activation of spleen pulp and terminal bronchopneumonia. Thromboemboli were found in several peripheral branches of the pulmonary artery. Hypertrophy of the heart (545 g), predominantly of the left ventricle, was also observed. The pancreas showed a macroscopically lobular arrangement and lipomatosis; other macroscopic changes were not visible. Lungs, thoracic lymph nodes, and other organs did not show any changes corresponding with tuberculous process or sarcoidosis.

## 3. Materials and Methods

Five representative tissue samples of pancreatic tissue taken from head, body, and tail were fixed with 10% formalin and routinely embedded in paraffin. Five-*μ*m-thick sections were stained with haematoxylin and eosin and by immunohistochemical methods using N-Histofine Immunohistochemical staining reagent (Nichirei Biosciences, Japan) and 3-3′diaminobenzidine as a chromogen to visualize the reaction. The list of antibodies, including manufacturers and the dilutions used, is introduced in Table 1.

## 4. Results

**Figure 1:**
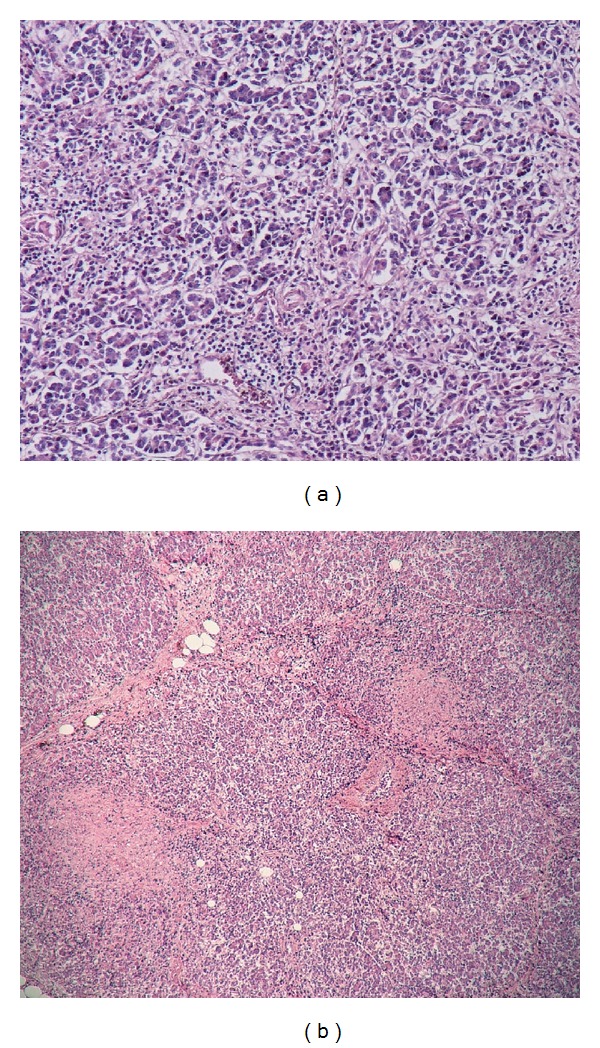
Focal inflammatory infiltrates within the pancreatic parenchyma. Haematoxylin and eosin, ×100 (a). Dispersed granulomas formed predominantly of epithelioid cells. Haematoxylin and eosin, ×40 (b).

**Figure 2:**
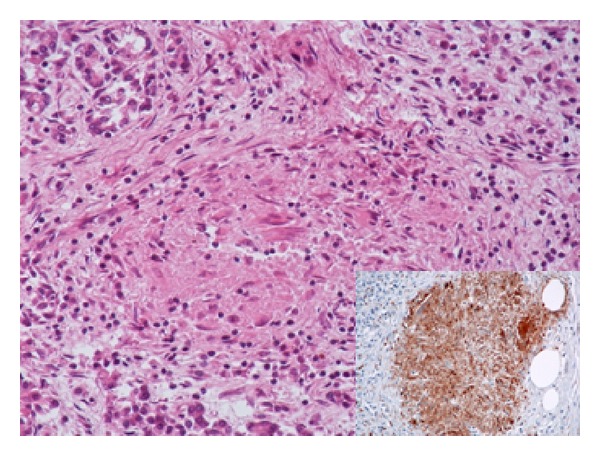
Epithelioid and giant cell granuloma. Haematoxylin and eosin, ×200. Inset: immunohistological detection of CD68 (B).

*Microscopic examination* of pancreatic tissue discovered an increased amount of lipomatous tissue within the pancreatic lobules. Irregular inflammatory infiltrates of a variable density composed predominantly of small lymphocytes and sparse neutrophilic and eosinophilic granulocytes were also observed (Figure 1(a)). Multiple dispersed small (up to 500 *μ*m) noncaseating epithelioid granulomas with giant cells, without Schaumann bodies, were present within the pancreatic lobules (Figures 1(b) and 2). Pancreatic islets were not found. Other organs examined histologically, that is, lungs, kidneys, and liver, did not display any granulomatous changes.

**Figure 3:**
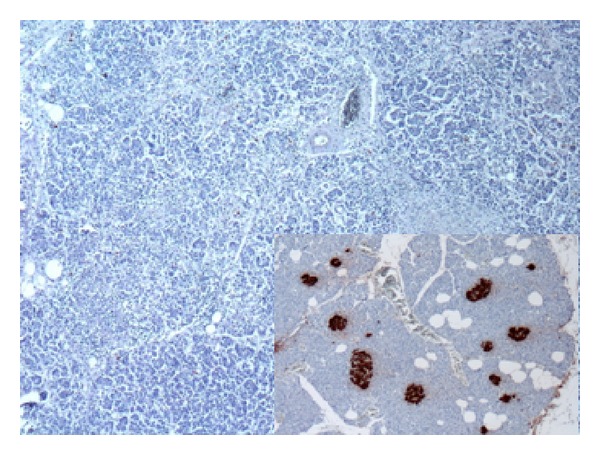
Absence of islets of Langerhans in the pancreatic tissue. Immunohistological detection of insulin, ×40. Inset: density of islets of Langerhans in normal (control) pancreatic tissue.

*Immunohistological examination* showed strong immunoreactivity of macrophages forming the granulomas for CD68 (Figure 2) and *α*-1 antichymotrypsin. Inflammatory infiltrates were composed predominantly of CD45R0 positive small T-lymphocytes and scattered CD20 positive small B cells. Immunohistological detection of markers of neuroendocrine differentiation (chromogranin A, synaptophysin) and pancreatic hormones (insulin, glucagon) verified the original microscopic finding of absence of islets of Langerhans (Figure 3).

## 5. Discussion

Granulomatous pancreatitis is a rare condition, infrequently described in infectious diseases, like tuberculosis or syphilis, and in systemic inflammations. Pancreatic tuberculosis is clinically nonspecific. Radiological findings can resemble the neoplastic process or chronic inflammation. Morphologically, it is characterized by caseating granulomas; caseous necrosis can be observed even in fine needle aspiration cytology [4, 5]. Syphilitic pancreatitis is an acquired disease extremely rare. It is manifested in the tertiary syphilis. Clinically jaundice, vague epigastric discomfort, diarrhoea, and fatigue can appear. In the microscopic examination, apart from noncaseating granulomas, vasculitis can also be observed [1, 2].

Noninfectious granulomatous pancreatitis can be observed in patients with systemic granulomatous diseases. In sarcoidosis, pancreatic involvement is rare. Clinically it is usually manifested as a mass resembling neoplasia [6–9]. Exceptionally, pancreatic sarcoidosis can be manifested by hypercalcemic pancreatitis [10]. Acute pancreatitis and diabetes mellitus have been also described in individual cases of pancreatic sarcoidosis [11]. The microscopic picture is characterized by noncaseating giant cell granulomas without accompanying lymphocytic reaction. Schaumann bodies, shell-like lamellated calcifications, are present in the giant multinuclear cells [1].

Focal granulomatous inflammation of pancreas has been described in Crohn's disease. Granulomas were noncaseating, contained numerous giant cells, and caused destruction of pancreatic tissue. Obstruction of the common bile duct clinically manifested with symptoms of extrahepatic cholestasis was present as a complication of this inflammatory process [12]. Foreign body (suture) granulomas can appear especially in the peripancreatic tissue as a consequence of prior surgery. These granulomas are typically composed of multinucleate or even polynucleate cells containing foreign birefringent material within the cytoplasm [1]. Exceptionally, granulomatous reaction is oriented to arteries. Isolated granulomatous arteritis can lead either to complete obliteration of the vessel or to thickening of fibrotic intima and narrowing of the lumen [1]. Granulomatous inflammation with foamy lipid-laden macrophages was observed in several cases of granulomatous pancreatitis and in experimentally induced insulitis in mice immunized with purified porcine insulin [1, 13].

Epithelioid cell granulomas, usually in ductulocentric location, have been rarely described in histological specimens of autoimmune pancreatitis (AP). The majority of patients were presented by the obstructive jaundice, weight loss, and abdominal pain. Other autoimmune disorders, like sclerosing cholangitis or interstitial pneumonia, can appear in patients with AP. Histologically, AP is characterized by dense lymphoplasmacytic infiltrates and secondary fibrosis within the pancreatic tissue. Inflammation frequently displays a patchy collar arrangement around both small and large interlobular ducts and periphlebitis and obliterative phlebitis is invariably observed [14]. It seems likely some previously described cases of isolated [3] or cryptogenic [1] granulomatous pancreatitis fulfill the diagnostic criteria for AP and clinically also correspond with this disorder. On the other hand, the histological picture of our case differs from the cardinal diagnostic features of AP.

Diabetes mellitus in adults is predominantly of type 2. Much less frequently, type 1 diabetes and latent autoimmune diabetes (LADA) can appear in adult patients [15]. Diabetes of the patient described in our report was originally clinically classified as LADA; however, ketoacidotic manifestation of the disease and requirement of urgent insulin treatment suggest acute manifested insulin-dependent diabetes mellitus corresponding with ketosis-prone diabetes (KPD) [16]. Granulomatous inflammation combined with disappearance of the islets has not been described so far, either in patients displaying characteristics of LADA or classical type 1 diabetes. Granulomatous lesions in the pancreas connected with diabetes were described under the experimental conditions in rats [17] and in one case of pancreatic sarcoidosis [11].

Our recent finding suggests that granulomatous pancreatitis is a possible underlying cause of diabetes mellitus and urges the microscopic examination of pancreatic tissue obtained during the post mortem examination of patients who died with signs and symptoms of recently manifested diabetes mellitus.

## Figures and Tables

**Table 1:** Antibodies and their dilutions used in the study.

Antibody	Source	Supplier	Dilution
Anti-insulin	Mouse monoclonal	Diagnostic Biosystems	1 : 50
	clone E2E3		
Anti-CD45R0	Mouse monoclonal	DAKO Cytomation	1 : 200
	clone UCHL 1		
Anti-CD20cy	Mouse monoclonal	DAKO Cytomation	1 : 100
	clone L26		
Anti-alpha-1	Mouse monoclonal	Acris Antibodies	1 : 400
Antichymotrypsin	clone ACT14C7		
Anti-CD68	Mouse monoclonal	DAKO Cytomation	1 : 100
	clone PG-M1		
Antichromogranin A	Rabbit polyclonal	DAKO Cytomation	1 : 600
